# Quantifying Coordination between Agonist and Antagonist Elbow Muscles during Backhand Crosscourt Shots in Adult Female Squash Players

**DOI:** 10.3390/ijerph18189825

**Published:** 2021-09-17

**Authors:** Abdel-Rahman Akl, Amr Hassan, Helal Elgizawy, Markus Tilp

**Affiliations:** 1 Faculty of Physical Education-Abo Qir, Alexandria University, Alexandria 21913, Egypt; abdelrahman.akl@alexu.edu.eg (A.-R.A.); helal.elgizawy@alexu.edu.eg (H.E.); 2 Department of Sports Training, Faculty of Sports Education, Mansoura University, Mansoura 35516, Egypt; amrahh@mans.edu.eg; 3 Institute of Human Movement Science, Sport and Health, University of Graz, A-8010 Graz, Austria

**Keywords:** racket sport, injury, elbow, electromyography, co-activation

## Abstract

The purpose of this study was to quantify the coordination between agonist and antagonist elbow muscles during squash backhand crosscourt shots in adult female players. Ten right-handed, international-level, female squash players participated in the study. The electrical muscle activity of two right elbow agonist/antagonist muscles, the biceps brachii and triceps brachii, were recorded using a surface EMG system, and processed using the integrated EMG to calculate a co-activation index (CoI) for the preparation phase, the execution phase, and the follow-through phase. A significant effect of the phases on the CoI was observed. Co-activation was significantly different between the follow-through and the execution phase (45.93 ± 6.00% and 30.14 ± 4.11%, *p* < 0.001), and also between the preparation and the execution phase (44.74 ± 9.88% and 30.14 ± 4.11%, *p* < 0.01). No significant difference was found between the preparation and the follow-through phase (*p* = 0.953). In conclusion, the co-activation of the elbow muscles varies within the squash backhand crosscourt shots. The highest level of co-activation was observed in the preparation phase and the lowest level of co-activation was observed during the execution. The co-activation index could be a useful method for the interpretation of elbow muscle co-activity during a squash backhand crosscourt shot.

## 1. Introduction

The popularity of squash is increasing and now it is one of the racket sports that is played in most countries in the world. Similarly, the number of squash studies is growing, together with the interest of scientists who have analyzed various aspects of the game [1,2,3,4]. 

Modern squash is a fast-performing sport including complex and multidirectional movement patterns with a high density and intermittent rhythm. Therefore, it is multifaceted in its motor skills and its physiological, kinetic, and cognitive requirements. Performance success depends to a large extent on the interaction and complementarity of these factors [3,5,6,7]. 

Previous studies analyzed the effect of upper extremity movement and racquet speed during skill performance in squash [8], examined the electromyographical activity during strokes [9], and performed three-dimensional kinematic analyses of the forehand [10] and backhand strokes [11]. 

In their study, Hong, Chang, and Chan [10] reported interesting differences in the types of skills used during a squash game, among them the fact that the backhand was played more frequently (63.1%) than the forehand (36.9%), which underlines the importance of the backhand stroke in squash. Others investigated the association between the rotating motion of the upper extremities and racket speed when playing squash., The rate of performance of the forehand and backhand stroke was similar in other studies [8,9,12]. 

The backhand in squash is different from other strokes because the player must control the racket to fully control the angle of the hit without losing control of the swing speed while controlling the angular displacements of the elbow, torso, and shoulder joints [12].

Seoung Eun, Seung Nam, and Murali [12] used a 3D motion analysis system to analyze the backhand stroke performance of both elite and novice players. The study aimed to compare the displacement and velocity of the trunk and racquet, and the angular displacements and the velocities of the elbow and shoulder joints. The significant differences observed between novice and expert players underline the importance of studying the muscular activity during the backhand stroke. Vukovic et al. [13] measured the trajectory and velocity of movement using a tracking system to determine whether there were significant differences between winners and losers. They analysed the used skills, the time patterns, and the position of the squash players during their performance. Subsequently, they compared the dynamic movements of players of different technical abilities and related them to the tactics adopted by different players in 24 competitive matches with elite male squash players [14]. McGarry [15] examined the space–time patterns of squash players as they move around the squash court in the context of a dynamical system using movements analysis of forty-eight squash rallies—twelve from each quarter-final match in a high-level knock-out competition.

Besides the analysis of performance, squash injuries have also been a focus for researchers. Finch and Eime [7] conducted a review on retrospective studies of squash injuries that analyzed records of hospitalized, injured, or emergency patients, and surveys from squash players. The studies included data from 2232 domestic league players and university teams from the USA, UK, New Zealand, Germany, and the Netherlands and concluded that better-controlled studies are needed, particularly to determine the risk of injuries associated with squash. 

In recent years, there have been steady increases in the duration of the game, perhaps due to the improvement in the physical and technical abilities of squash players. In addition, it should be noted that in 2009 squash underwent changes in the rules (e.g., changing the scoring system in squash to ‘Point-A-Rally’ (PAR) to 11 points per game) according to which players have less time to perform shots, which increases the workload of the players [14]. Both developments may cause high loads leading to injury.

Therefore, an important research aim in sports medicine has been to understand the relationship between the intensity or volume of training and the type and grade of injuries mainly due to overuse, particularly following stroke training [16,17,18]. During the last few years, some researchers have focused on injuries in squash [18]. Horsley et al. [19] studied the diversity of injuries suffered by professional squash players in both training and competition through a survey of injury records between 2004–2015. However, this study only looked at injuries of the lower limbs because of the mechanical loading of the players during strokes in racket games. Habitual participation in racquet games over years often results in specific strength and flexibility imbalances [20]. Previous studies have reported that the ratio of upper-limb injuries is around 36% of all squash injuries, making the elbow the most commonly injured body region [3,5,7]. Despite this, little is known about injury mechanisms, exhaustion, recovery, and performance in training and competition [21,22,23].

Previous studies have indicated the relationship between muscular co-activation and injury. Hirokawa, et al. [24] reported that increased quadriceps–hamstring muscle co-activation at the knee may reduce the risk of anterior cruciate ligament (ACL) injury.

Lehman [25] reported that there is a relationship between muscle extensor endurance with decreases in injury risk. The aberrant flexor/extensor endurance ratios have also been correlated with a history of injury. From this perspective, adequate joint stability is related to the amount of muscle co-activation [26,27]. Elbow stability is not provided by one specific muscle but rather via the coordinated efforts of agonist and antagonists muscles. These muscles are active throughout the whole backhand crosscourt movement in squash. Due to the muscular demands of the backhand crosscourt shot and the prevalence of injury in the elbow, training the agonist and antagonist musculature may improve performance and decrease injury risk.

Surprisingly, so far, no study has investigated the electrical activity of the muscles during the backhand stroke, which has been identified as the skill with the highest frequency and also the highest cause of injury rate [3,7]. 

Given the importance of the coordination between the muscles working on the elbow joint with regards to performance and injury prevention, especially among female athletes, this study aims to investigate the coordination between agonist and antagonist elbow muscles during the backhand crosscourt stroke in adult female squash players.

## 2. Materials and Methods

### 2.1. Participants

Female, right-handed elite squash players (*n* = 10) participated in the present study (age: 18.4 ± 0.8 years; body mass: 60.8 ± 1.8 kg; height: 165.2 ± 1.6 cm; training age: 9.1 ± 0.9 years). The subjects were officially ranked between 4 and 20 in the Egyptian squash federation and were currently competing in professional squash tournaments (national and international). Written informed consent of the players was obtained, and the study was approved by the institutional ethics committee of studies and research. 

### 2.2. Experiment Protocol

After a 15 min warm-up including general, elbow-, and shoulder-specific mobility exercises, as well as stretching and familiarization with the protocol, participants performed Squash backhand crosscourt strokes. A total of three successful attempts were recorded for each player, with a one-minute rest between attempts. The Squash backhand crosscourt skill was broken into three phases: the preparation phase, the execution phase, and the follow-through phase.

### 2.3. Data Recording

The electrical muscle activity of two right elbow agonist/antagonist muscles, the biceps brachii (BB) and triceps brachii (TB) were recorded using surface EMG system (Myon m320RX; Myon, Switzerland). The skin over the muscles of the dominant arm was shaved and cleaned with alcohol and bipolar, circular 10 mm diameter silver chloride surface electrodes (SKINTACT FS-RG1/10, Leonhard Lang GmbH, Archenweg 56, 6020 Innsbruck, Austria) were secured on the selected muscles. Electrodes were attached over each muscle following the SENIAM guidelines maintaining a 2 cm center to center inter-electrode spacing [28]. The EMG signals were stored at a sampling frequency of 1000 Hz and digitized using a 16-bit analog to digital (A/D) converter. EMG data was processed using Visual 3D software (C-Motion, Germantown, MD, USA). Raw EMG data were band-pass filtered (20 Hz–450 Hz) applying a Butterworth filter. The signals were preprocessed using full wave rectifier and a linear envelope obtained using the root mean square (RMS) approach with a window size of 100 ms. Data were normalized to an isometric maximum voluntary contraction (MVC), which was recorded after each subject finished the experimental tasks. To obtain the MVC values, subjects performed three repetitions for 5 s, with 60 s rest in between while sitting in a stable chair with forearm resistance. Peak muscle activity over the three repetitions for each muscle was taken as the MVC value.

### 2.4. Co-Activation Index 

**Figure 1:**
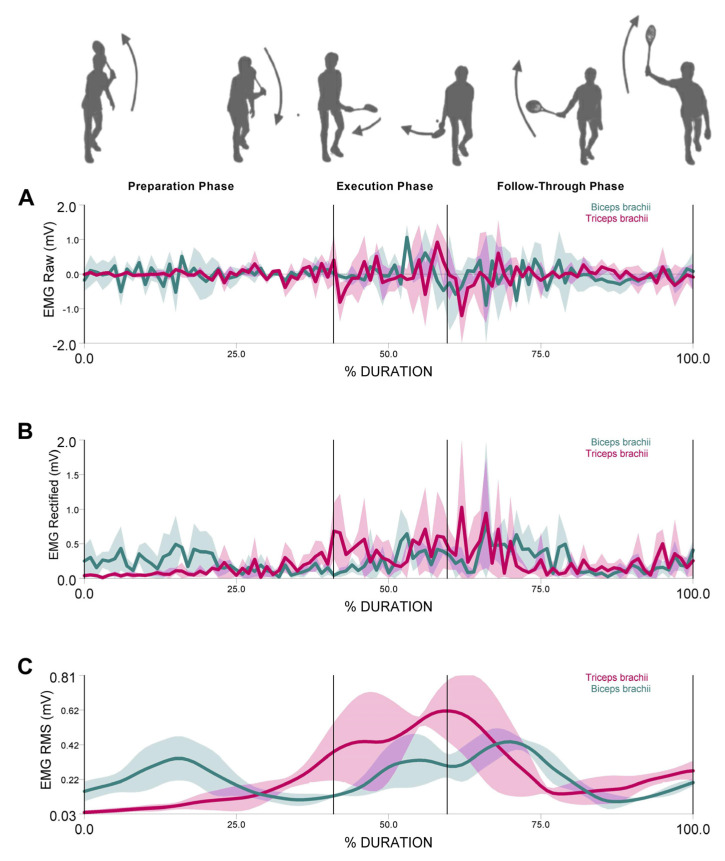
Backhand crosscourt phases (Preparation phase, Execution phase, Follow-Through phase) of the Biceps brachii (BB) and Triceps brachii (TB). (**A**) EMG raw data, (**B**) EMG rectified data, and (**C**) EMG RMS. Means (solid lines) and standard deviation (shaded areas) of three stroke attempts from a representative subject.

Muscle co-activation was estimated by the calculation of a co-activation index (CoI) using the following equation adapted from Kellis et al. [29]

$$CoI=\frac{iEMG_{anta}}{\left(iEMG_{anta}+iEMG_{ago}\right)}\;\times 100$$

where *iEMGanta* and *iEMGago,* respectively, refer to *iEMG* of antagonist and agonist muscle in different movement phases. The preparation phase was defined from the beginning of the movement to the end of the elbow flexion, the execution phase was defined from the beginning of the elbow extension until the shot, and the follow-through phase was defined from the instant of the shot until the end of the movement, see Figure 1. The phases were defined by video analysis using 3D simi motion capture, which was synchronized with EMG.

### 2.5. Statistical Analysis 

Descriptive statistics were reported as means and standard deviations (mean ± SD). The normality of the data was analyzed using the Shapiro–Wilk test and all data were found to be suitable for parametric analysis. Repeated Measures Analysis of Variance (ANOVA) with Sidak post hoc tests were used to detect significant differences and compare the mean of each variable during the three phases (preparation, execution, and follow-through). Partial eta squared (η^2^p) was calculated to assess the effect size. The statistical analysis was performed using IBM SPSS software Statistics v21 (IBM^®^ Corporation, Armonk, NY, USA).

## 3. Results

### 3.1. Muscular Activity

**Figure 2:**
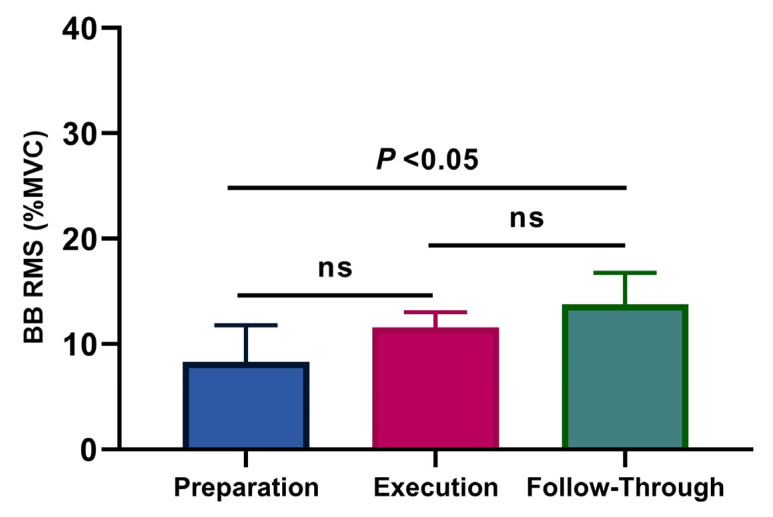
Average values and standard deviations for the normalized EMG (%MVC) per phase of the biceps brachii (η^2^p = 0.50).

**Figure 3:**
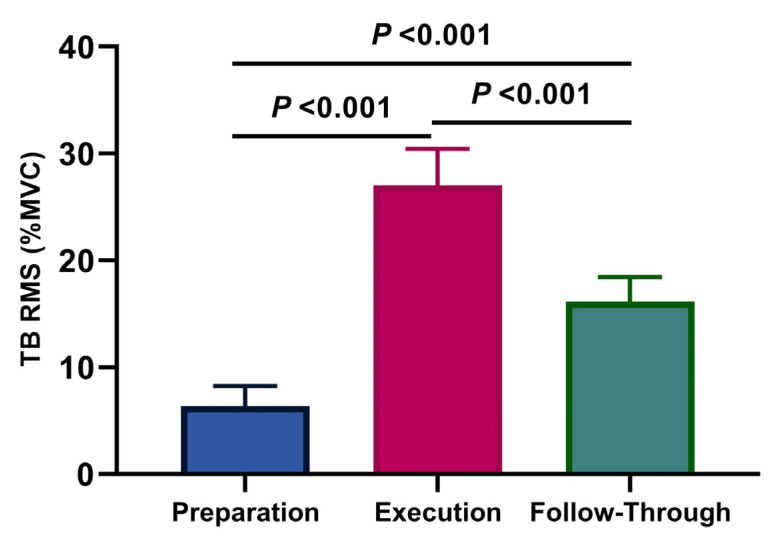
Average values and standard deviations for the normalized EMG (%MVC) per phase of the Triceps brachii (η^2^p = 0.97).

Average values and standard deviations for the normalized RMS for the BB are presented in Figure 2, and the TB in Figure 3, during the three analyzed phases (the preparation, the execution, and the follow-through phase). The highest activities of the BB were observed during the follow-through phase, followed by the execution phase, and the preparation phase, with values of 13.80 ± 2.97%, 11.57 ± 1.45%, and 8.32 ± 3.47%, respectively. There was a significant difference between the BB activity during the preparation compared to the follow-through phases (*p* < 0.05; η^2^p = 0.50, Figure 2). For TB, the highest activities were observed during the execution phase, followed by the follow-through phase and the preparation phase, with values of 27.02 ± 3.43%, 16.14 ± 2.32%, and 6.38 ± 1.86%, respectively, while high significant differences for the TB were observed among the three phases (*p* < 0.001; η^2^p = 0.97, Figure 3). 

### 3.2. Co-Activation Index

**Figure 4:**
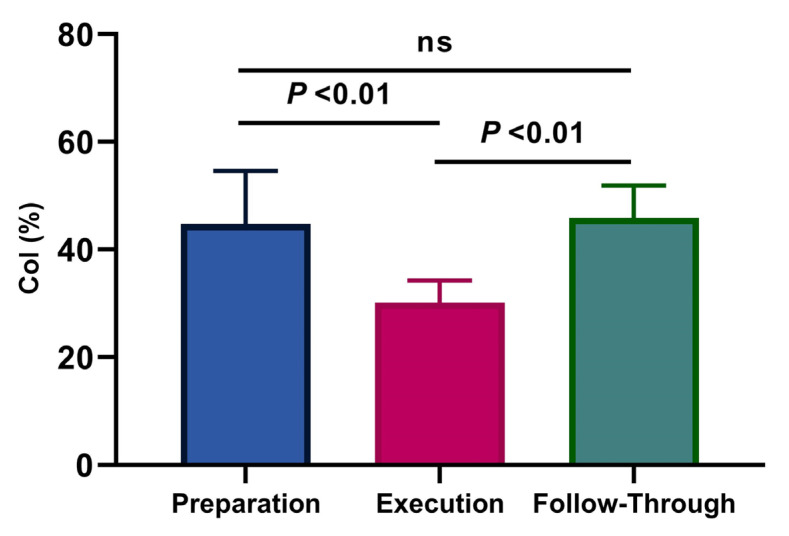
Average values and standard deviations for the co-activation index (CoI) (%) per phase (η^2^p = 0.73). The agonist/antagonist muscle of each phase was BB/TB, TB/BB, and TB/BB, respectively.

A significant effect (*p* < 0.01, η^2^p = 0.73) of the phases on the CoI was observed (Figure 4). Post hoc analyses showed that the co-activation was significantly higher in the follow-through phase compared to the execution phase (45.93 ± 6.00% and 30.14 ± 4.11%, *p* < 0.001), and also between the preparation phase and the execution phase (44.74 ± 9.88% and 30.14 ± 4.11%, *p* < 0.01). No significant difference was found between the preparation and follow-through phases (*p* = 0.95). 

## 4. Discussion

The main aim of this study was to determine muscle co-activation of elbow muscles as an indicator of coordination between agonist and antagonist muscle activity during three phases of the squash backhand crosscourt shots in adult female players. While we observed similar co-activation in the preparation and follow-through phase, the co-activation was significantly decreased during the execution phase. 

The main activities for the BB and the TB were observed during three phases in which they acted as a prime mover (agonist), BB during the preparation phase and TB during the execution and follow-through phases.

Low values of muscle activity were observed in the preparation phase where both BB and TB showed activations less than 10% of MVC (BB: 8.3% MVC and TB 6.4% MVC). This can be explained by the fact that the elbow flexion includes muscle synergies (e.g., brachioradialis and anterior deltoid muscle) [30,31]. However, the relative muscle activity of BB was greater than that of TB muscle activity because the BB is the prime mover during elbow flexion. 

Greater TB activation was observed during the execution phase with 27.02% MVC (BB: 11.57% MVC). Despite substantial activation differences between TB and BB during the execution phase, the observed BB activation was still greater when compared with the preparation phase. This is somewhat surprising, since the BB is an agonist muscle in the preparation phase but an antagonist muscle in the execution phase. The reason for this result may be the variation in movement muscle activity amplitude. While the agonist muscle activity increased during the execution phase to accelerate the movement, the antagonist initiated an increase in muscle activity, possibly to prevent an elbow joint injury due to overextension of the elbow joint in the follow-through phase [32].

Previous studies [33,34,35,36,37,38] indicate that experienced athletes could have a distinct muscle activation pattern with less antagonist muscle activation, implying that antagonistic muscle coupling might be altered by specialized activity. As a result, top athletes may have lower muscle co-activation than non-athletes, particularly during fast movements [39]. 

According to the observed results, muscle co-activation was greater in the preparation and the follow-through phases compared to the execution phase. Both Bazzucchi et al. [40] and Rouard and Clarys [31] reported greater co-activation values of the arm muscles during fast compared to slow movements, increasing at the preparation phase, decreasing at execution to allow faster acceleration, and increasing at the end of the movement to provide dynamic braking, which is similar to the elbow extension in the backhand crosscourt shot. In addition, Darainy and Ostry [41] and Bazzucchi, Sbriccoli, Marzattinocci, and Felici [40] underlined that greater antagonist activity could make the motor task controllable and also increases the stiffness and stability of the joint.

Wagner et al. [42] reported that overarm movements are essential skills in different types of sports. Hence, strong elbow extension might be considered as one of the determinants of efficiency in the squash backhand crosscourt shot. Bazzucchi, Riccio, and Felici [39] observed that muscle co-activation decreased during the execution phase for generating higher forces to increase performance. This is in accordance with our findings where the TB as the agonist muscle showed a strong activity during the execution phase, with low values of the BB as the antagonist muscle. This led to a low value of co-activation during the execution phase with values around 30%.

Muscle co-activation increased again in the follow-through phase to inhibit end range elbow extensions. The high value of co-activation in the follow-through phase with values of 45.93% increased joint stiffness and, therefore, stability [40]. This result was expected as increased co-activation is considered a determinant for preventing injury of the elbow joint. 

Thus, an initial decreased co-activation allows a faster acceleration in the execution phase, while an increase at the end of the movement range provides dynamic braking of the movement. Furthermore, the high co-activation at the end of the movement allows players to better prepare the arm for the next response during the game [30,40].

Whereas most previous research has concentrated on antagonist muscle co-activation during maximal isometric efforts or as a function of isokinetic velocity, our research focused on sports movements including both concentric and eccentric contractions [43].

## 5. Conclusions

In summary, the co-activation of the elbow muscles varies within the squash backhand crosscourt stroke. The highest level of co-activation was observed in the preparation phase to control the forearm velocity before the execution phase, and in the follow-through phase to stabilize the elbow joint to prevent injuries and slow down the arm at the end of the movement. The lowest level of co-activation was observed in the execution phase for generating the appropriate force from the prime mover muscle to increase the efficiency of the backhand crosscourt shot.

## Data Availability

The data presented in this study are available on request from the first author.
